# Immune cell expression patterns of CD39/CD73 ectonucleotidases in rodent models of cardiac arrest and resuscitation

**DOI:** 10.3389/fimmu.2024.1362858

**Published:** 2024-03-13

**Authors:** Tomoaki Aoki, Vanessa Wong, Tai Yin, Eriko Nakamura, Yusuke Endo, Kei Hayashida, Simon C. Robson, Harshal Nandurkar, Betty Diamond, Sun Jung Kim, Atsushi Murao, Ping Wang, Lance B. Becker, Koichiro Shinozaki

**Affiliations:** ^1^ Department of Emergency Med-Cardiopulmonary, The Feinstein Institutes for Medical Research, Northwell Health, Manhasset, NY, United States; ^2^ State University of New York Downstate Medical Center, NY, United States; ^3^ Department of Anesthesia, Beth Israel Deaconess Medical Center, Harvard Medical School, Boston, MA, United States; ^4^ Australian Centre for Blood Diseases, Monash University, Melbourne, VIC, Australia; ^5^ Institutes of Molecular Medicine, The Feinstein Institutes for Medical Research, Northwell Health, Manhasset, NY, United States; ^6^ Center for Immunology and Inflammation, The Feinstein Institutes for Medical Research, Northwell Health, Manhasset, NY, United States; ^7^ Department of Emergency Medicine, Donald and Barbara Zucker School of Medicine at Hofstra/Northwell Health, Hempstead, NY, United States; ^8^ Department of Emergency & Critical Care Medicine, Kindai University Faculty of Medicine, Osaka, Japan

**Keywords:** rodent, monocytes, T cells, B cells, heart arrest, cardiopulmonary resuscitation, ischemia, reperfusion injury

## Abstract

**Background:**

Cardiac arrest (CA) is a significant public health concern. There is the high imminent mortality and survival in those who are resuscitated is substantively compromised by the post-CA syndrome (PCAS), characterized by multiorgan ischemia–reperfusion injury (IRI). The inflammatory response in PCAS is complex and involves various immune cell types, including lymphocytes and myeloid cells that have been shown to exacerbate organ IRI, such as myocardial infarction. Purinergic signaling, as regulated by CD39 and CD73, has emerged as centrally important in the context of organ-specific IRI. Hence, comprehensive understanding of such purinergic responses may be likewise imperative for improving outcomes in PCAS.

**Methods:**

We have investigated alterations of immune cell populations after CA by utilizing rodent models of PCAS. Blood and spleen were collected after CA and resuscitation and underwent flow cytometry analysis to evaluate shifts in CD3^+^CD4^+^ helper T cells, CD3^+^CD8a^+^ cytotoxic T cells, and CD4/CD8a ratios. We then examined the expression of CD39 and CD73 across diverse cell types, including myeloid cells, T lymphocytes, and B lymphocytes.

**Results:**

In both rat and mouse models, there were significant increases in the frequency of CD3^+^CD4^+^ T lymphocytes in PCAS (rat, *P* < 0.01; mouse, *P* < 0.001), with consequently elevated CD4/CD8a ratios in whole blood (both, *P* < 0.001). Moreover, CD39 and CD73 expression on blood leukocytes were markedly increased (rat, *P* < 0.05; mouse, *P* < 0.01 at 24h). Further analysis in the experimental mouse model revealed that CD11b^+^ myeloid cells, with significant increase in their population (*P* < 0.01), had high level of CD39 (88.80 ± 2.05 %) and increased expression of CD73 (*P* < 0.05). CD19^+^ B lymphocytes showed slight increases of CD39 (*P* < 0.05 at 2h) and CD73 (*P* < 0.05 at 2h), while, CD3^+^ T lymphocytes had decreased levels of them. These findings suggested a distinct patterns of expression of CD39 and CD73 in these specific immune cell populations after CA.

**Conclusions:**

These data have provided comprehensive insights into the immune response after CA, highlighting high-level expressions of CD39 and CD73 in myeloid cells.

## Introduction

Cardiac arrest (CA) is a major public health issue, afflicting more than 356,500 people in the out-of-hospital setting and 209,000 people in the in-hospital setting in the United States each year (1). Post-CA syndrome (PCAS) is a lethal condition characterized by high mortality and systemic ischemia–reperfusion injury (IRI) (2–4), which can trigger immune responses that play a significant role in the pathophysiology after CA (5).

Even after successful resuscitation, out-of-hospital CA (OHCA) patients suffer from a hyperinflammatory status, a so called sepsis-like syndrome, which is characterized by high levels of circulating pro-inflammatory cytokines and a dysregulation of the immune system (6–9). Hyporesponsiveness of circulating leukocytes such as decreased production of cytokines in OHCA patients was observed ex vivo (8–10), indicating that the cell-mediated immunity of OHCA patients is impaired (10). These changes are also similar to those in sepsis, in which the immune system is disordered (11), therefore, understanding the mechanisms underlying this immune disorder may be the key to rescuing OHCA patients after return of spontaneous circulation (ROSC).

The immune response during and after CA and resuscitation is a complex process that involves various immune cell types, and understanding their roles is crucial for improving survival. Cluster-of-differentiation 3 (CD3) is a cell surface protein complex found on T cells, which are a type of leukocyte that plays a central role in the immune system. CD4 is a glycoprotein found on the surface of helper T cells. Helper T cells play a vital role in orchestrating the immune response by assisting other immune cells in recognizing and responding to pathogens or damaged tissues. CD8a is found on cytotoxic T cells, which recognize and eliminate infected or damaged cells. Although these lymphocytes assume a central role in the immune response, differential counts of these T cell populations after CA have been scarcely addressed. In addition to T lymphocytes, other major immune cells expressing CD19 or CD11b are also involved in post-CA immune responses. CD19 is found primarily on B cells, a type of leukocyte involved in the immune response that can become relevant following acute phase of diseases that are associated with infection, inflammatory conditions, or other conditions triggering a systemic inflammatory response (12). CD11b is an integrin receptor primarily found on the surface of myeloid cells, specifically neutrophils and monocytes. These myeloid cells are important components of the immune system and play a role in the systemic response from injuries, infection, and inflammation, specifically in the adhesion of these immune cells to the blood vessel wall, their subsequent migration into tissues, and neutrophil activation (13, 14).

In addition to aforementioned surface markers characterizing major immune cell populations, we explored the intricate field of purinergic signaling, a novel focal point in the context of IRI (15). Elucidating this pathway holds paramount significance in mitigating hyperinflammatory responses and enhancing post-CA survival.

Adenosine triphosphate (ATP), a fundamental molecule for intracellular energy transfer, assumes a dual role in which, when released from ischemic cells into the extracellular milieu, purines become signaling molecules. Extracellular ATP (eATP) is capable of activating purinergic receptors, specifically P2X and P2Y receptors, thereby triggering cellular injury and hyperinflammatory cascades, leading to cell death and organ dysfunction (16, 17). eATP can be converted stepwise to adenosine by cell-surface enzymes such as ectonucleoside triphosphate diphosphohydrolase-1 (ENTPD1, CD39) and ecto-5'-nucleotidase (NT5E, CD73). Nucleotide phosphohydrolysis into adenosine is a multi-step process facilitated by these ectoenzymes. CD39 catalyzes the conversion of adenosine tri- and diphosphates to monophosphate, while CD73 further converts the monophosphate into adenosine. Notably, when adenosine binds to purinergic receptors, it exerts cytoprotective and anti-inflammatory effects (16, 17). Both CD39 and CD73 hold the potential to significantly impact the outcomes of CA and resuscitation through the fine-tuned regulation of adenosine levels.

In this study, cytometric analyses to assess alterations in predominant immune cell populations and expression of ectonucleotidases following CA were investigated. We employed both rat and mouse models of CA and PCAS.

## Materials and methods

Animal protocols were approved by The Institutional Animal Care and Use Committees of Feinstein Institutes for Medical Research (#2016-004, and #2022-012). All methods were performed in accordance with the National Institutes of Health Guide for the Care and Use of Laboratory Animals and ARRIVE guidelines.

### Rat cardiac arrest animal preparation

**Figure 1:**
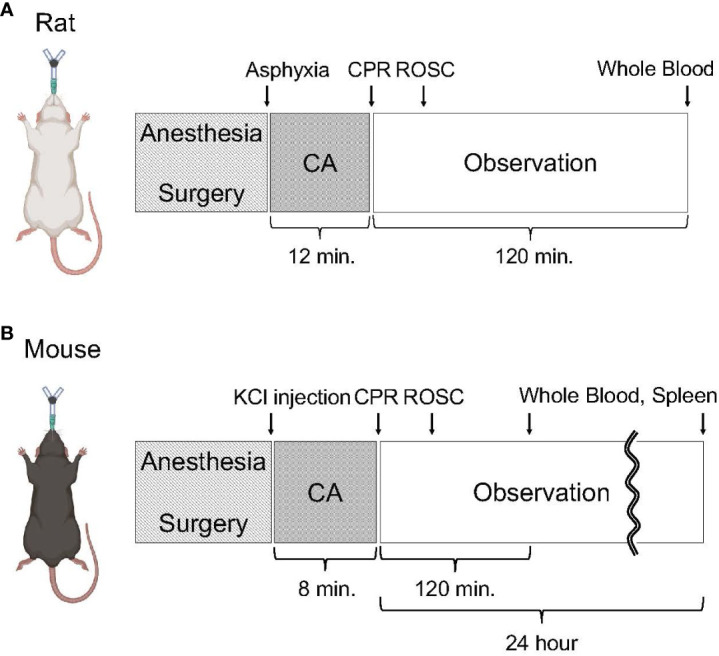
Experimental protocol on animal surgery. **(A)** Animal protocol of rat CA. Rats were subjected to 12 minutes of asphyxia-induced CA and resuscitation. Following observation under mechanical ventilation for 120 minutes after initiating CPR, whole blood was collected. **(B)** Animal protocol of mouse CA. Mice were subjected to 8 minutes of KCl-induced CA and resuscitation. At 2 hours or 24 hours after initiating CPR, whole blood and the spleen were collected.

We performed all instrumentation according to the previously described protocol (**Figure 1A**) (4, 18, 19). In brief, adult male 12-16-week-old Sprague-Dawley rats (400–500 g, Charles River Laboratories, Wilmington, MA, USA) were anesthetized with 4% isoflurane (Isosthesia, Butler-Schein AHS, Dublin, OH, USA) and intubated with a 14-gauge plastic catheter (Surflo, Terumo Medical Corporation, Tokyo, Japan). We used only male rats to reduce potential confounding among animals that may cause outcome variabilities from hormonal and/or genetic differences. Rats were mechanically ventilated (Ventilator Model 683, Harvard Apparatus, Holliston, MA, USA) at a minute ventilation (MV) volume of 180 mL per minute at a respiratory rate of 45 breaths per minute. In this study, we used one ventilation setting for all animals at all times and did not change the MV or respiratory rate during the experiments. Positive end-expiratory pressure (PEEP) was set at 2 cm H_2_O. Anesthesia was maintained with isoflurane at 2% and at a fraction of inspired O_2_ (F_I_O_2_) of 0.3. Core temperature was maintained at 36.5 ± 1.0 °C during the surgical procedure. After instrumentation, neuromuscular blockade was achieved by slow intravenous administration of 2 mg/kg of vecuronium bromide (Hospira, Lake Forest, IL, USA) and asphyxia was induced by turning off the ventilator. CA normally occurred three to four minutes after asphyxia started. The CA group animals received cardiopulmonary resuscitation (CPR) after they were subjected to 12 minutes asphyxia. We defined CA as a mean arterial pressure (MAP) of < 20 mmHg; CA was completely untreated during the initial 10 minutes. After 12 minutes of asphyxia, mechanical ventilation was restarted at an F_I_O_2_ of 1.0 and manual chest compression CPR was delivered simultaneously. Chest compressions were performed with two fingers over the sternum at a rate of 260 to 300 per minute. At 30 seconds after beginning of CPR, a 20 µg/kg bolus of epinephrine was given to rats through a venous catheter. Following ROSC, defined as MAP > 60 mmHg, CPR was discontinued. At 2 hours after initiating CPR, mechanical ventilation was discontinued, and rats were euthanized for sample collections of whole blood. Whole blood were drawn through the arterial catheter.

### Mouse cardiac arrest animal preparation

We performed all instrumentation according to the previously described protocol (**Figure 1B**) (20). In brief, adult male 14-18-week-old C57BL/6 mice (weight 25 to 30 g; Charles River Production, Wilmington, MA, USA) were freely accessed to food and water before experiments. Mice were anesthetized with 4% isoflurane (Isosthesia, Butler-Schein AHS, Dublin, OH, USA) and the trachea was orally intubated with a 20-gauge catheter (Angiocath, Becton Dickinson). Mechanical ventilation (Minivent, Harvard Apparatus, Holliston, MA, USA) was initiated at rate of 110/min, I:E = 1:1, F_I_O_2_ = 1.0, and tidal volume was maintained at 8–12 mL/kg per minute. We used only male mice to reduce potential confounding among animals that may cause outcome variabilities from hormonal and/or genetic differences. In this study, we used one ventilation setting for all animals at all times and did not change the MV or respiratory rate during the experiments. PEEP was set at 1 cm H_2_O. Anesthesia was maintained with isoflurane at 2%. During surgical preparation, body temperature was maintained between 37.0 ± 0.5 °C by an incandescent heating lamp and monitored by a transesophageal thermocouple probe. A microcatheter (EZ-1101, BioTime Inc, Carlsbad, CA, USA) was inserted into the left femoral vein for drug and fluid administration. The other microcatheter was inserted into left femoral artery to monitor the blood pressure. Electrocardiograph (ECG) was recorded by needle probe. The mouse CA was quickly induced by 100 μL KCl (80 μg/kg) followed by 200 μL room temperature saline flushing and weaned off the ventilator at the same time. The CA was confirmed by loss of ECG activity and blood pressure, which usually sharply decreased, below 10 mmHg. The esophageal temperature of the mice was maintained at 37.0 ± 0.5 °C during the whole procedures by heating lamp. Eight minutes after the induction of CA, resuscitation via manual chest compression with one finger over the sternum at a rate of 300 to 400 per minute was initiated together with 1 μg/100 μL epinephrine followed by flushing with 200 μL room temperature saline via left femoral vein, and ventilation was resumed at the rate of 110/min, I:E = 1:1, F_I_O_2_ = 1.0 with tidal volume maintained at 8–12 mL/kg per minute. Chest compression was performed to provide a uniform rate and to maintain maximal MAP. ROSC was confirmed by the return of ECG rhythm and significant increase in MAP from that during chest compression. The mouse was maintained on mechanical ventilation after ROSC. At 2 hours after initiating CPR, mechanical ventilation was discontinued, and mice were euthanized for sample collections of the spleen and whole blood. For the assessment of alterations in predominant immune cell populations and the expression of ectonucleotidases at a later time point, 3 mice were monitored until 24 hours after initiating CPR (**Figure 1B**). Subsequent to extubation, a subcutaneous injection of 0.05 mL extended-release buprenorphine (Ethiqa XR, Fidelis Pharmaceuticals, North Brunswick Township, NJ, USA) was administered to the animals for analgesia. At 24 hours after initiating CPR, the mice were euthanized for sample collections of the spleen and whole blood. Whole blood samples were obtained from inferior vena cava under inhaled isoflurane anesthesia prior to perfusion and subsequent spleen extraction. Postsurgical care, including animal housing and observation, were provided by the animal facility.

### Cell preparation of whole blood for flow cytometry

Whole blood samples were collected into heparinized tubes. In the rat CA experiment, 500 μL of whole blood was collected after catheterization as baseline and again at 2 hours after initiating CPR. In the mouse CA experiment, animals were euthanized 2 hours or 24 hours after initiating CPR and 500 μL of whole blood was collected, and 500 μL of whole blood was collected from a naïve mouse for reference purposes. The collected blood samples were diluted into 5 mL of 1× RBC Lysis Buffer (prepared from 10× RBC Lysis Buffer, BioLegend, San Diego, CA, USA). After an incubation period of 4-5 minutes at room temperature with occasional shaking, the reaction was halted by dilution of the Lysis Buffer with 20-30 mL of 1× PBS. Subsequently, cells were pelleted by centrifugation at 500×g at room temperature for 5 minutes and then resuspended in 2 mL of 1× RBC Lysis Buffer. Following a similar incubation duration with occasional shaking, the reaction was halted by diluting the Lysis Buffer with 10-15 mL of 1× PBS. Cells were pelleted again by centrifugation at 500×g at room temperature for 5 minutes and finally resuspended in 500 μL of Flow Cytometry Staining Buffer (Invitrogen, Waltham, MA, USA).

### Cell preparation of the spleen for flow cytometry

In the mouse CA experiment, a spleen was harvested 2 hours or 24 hours after initiating CPR, meanwhile a spleen from a naïve mouse was harvested for comparative reference. The harvested spleen sample was finely minced using sharp scissors and then placed onto a 70 μm Cell Strainer (Corning, NY, USA), which was situated atop a 50 mL tube. The minced spleen was further homogenized using the plunger end of a 5 mL syringe and rinsed with 5 mL of 1× PBS. Subsequently, the strained cells were pelleted by centrifugation at 500×g at room temperature for 5 minutes. The resulting cell pellets were resuspended in 2 mL of 1× RBC Lysis buffer. After an incubation period of 1-2 minutes at room temperature with occasional shaking, the reaction was halted by dilution of the Lysis Buffer with 10-15 mL of 1× PBS. Subsequently, cells were pelleted by centrifugation at 500×g at room temperature for 5 minutes and finally resuspended in 500 μL of Flow Cytometry Staining Buffer.

### Microscale protein labeling

A single fluorophore was conjugated to either the anti-rat CD39/ENTPD antibody (Proteintech, cat no. 14211-1-AP, Rosemont, IL, USA) or the anti-rat NT5E/CD73 antibody (Proteintech, cat no. 12231-1-AP) using the Alexa Fluor® 594 Microscale Protein Labeling Kit or the Alexa Fluor® 647 Microscale Protein Labeling Kit (Invitrogen), respectively, following the manufacturer’s provided protocols. Briefly, 1 M sodium bicarbonate solution was prepared by adding 1 mL deionized water to the vial of sodium bicarbonate (Component B). Next, 50 µL of antibodies and 5 µL of Component B were transferred to a designated reaction tube (Component C). Separately, 10 µL of deionized water was introduced to each vial containing Alexa Fluor 594 and 647 succinimidyl ester (Component A). Subsequently, 0.616 µL (Alexa Fluor 594) or 0.7 µL (Alexa Fluor 647) of Component A was transferred to Component C, followed by a 15-minute incubation period at room temperature. To purify the Alexa Fluor 594-labeled anti-rat CD39 antibody and the Alexa Fluor 647-labeled anti-rat CD73 antibody, 50 µL of Component C was meticulously dispensed onto the center of the resin bed surface within specialized spin columns. The spin columns were then subjected to centrifugation at 16,000×g for 1 minute, effectively yielding the purified labeled antibodies in the collection tubes.

### Flow cytometry analysis

Single-cell suspensions derived from whole blood samples in both the rat and mouse experiments, as well as from the spleen in the mouse experiment, were subjected to flow cytometry analysis. This analysis involved the use of fluorophore-conjugated antibodies (Thermo Fisher Scientific, Waltham, MA, USA) targeting specific cell surface markers, including anti-rat CD3-PE (eBioScience, clone# eBioG4.18), anti-rat CD4-FITC (eBioScience, clone# OX35), anti-rat CD8a-APC (eBioScience, clone# OX8), anti-mouse CD3-APC-eFluor™ 780 (eBioScience, clone# 17A2), anti-mouse CD4-FITC (eBioScience, clone# GK1.5), anti-mouse CD8a-APC (eBioScience, clone# 53-6.7), anti-rat CD39-Alexa Fluor 594 (manually labeled), anti-rat CD73-Alexa Fluor 647 (manually labeled), anti-mouse CD45-PerCP-Cyanine5.5 (eBioScience, clone# 30-F11), anti-mouse CD39-PE-Cyanine7 (eBioScience, clone# 24DMS1), rat IgG2b isotype control-PE-Cyanine7 (eBioScience, clone# eB149/10H5), anti-mouse CD73-PE (eBioScience, clone# eBioTY/11.8), rat IgG1 isotype control-PE (eBioScience, clone# eBRG1), anti-mouse CD11b-FITC (eBioScience, clone# M1/70), and anti-mouse CD19- eFluor™ 450 (eBioScience, clone# eBio1D3). To reduce Fc receptor-mediated binding by antibodies of interest for analyses of both myeloid cells and B lymphocytes, cell suspension were preincubated with anti-mouse CD16/CD32 (BD Biosciences, clone# 2.4G2) at 4˚C for 5 minutes. The cells were incubated with a mixture of these antibodies at saturating concentrations in Flow Cytometry Staining Buffer at room temperature in darkness for 30 minutes. Following this incubation period, the cells were stained with 4’,6-diamidino-2-phenylindole (DAPI, cat no. D1306) for the exclusion of dead and apoptotic cells from flow cytometric analysis and then carefully washed. Subsequently, they were resuspended in 500 μL of Flow Cytometry Staining Buffer. Data were acquired on BD Symphony (BD Biosciences) and subsequently analyzed using FlowJo software 10.8.1 (Becton, Dickinson & Company, Ashland, OR, USA).

### Statistical analysis

Data are shown as means and standard errors of the means (SEM) for all variables. Two-tailed *P*-values of Wilcoxon matched-pairs signed rank test and Mann-Whitney test were used for two-group comparisons: between baseline and 2 hours after CA in the rat model; and between naïve and 2 hours after CA in the mouse model, respectively. Dunn’s multiple comparisons test was used for three-group comparisons: between naïve, 2 hours, and 24 hours after CA in the mouse model. *P <* 0.05 was considered statistically significant. Prism 10.0.3 (GraphPad, San Diego, CA, USA) was used for these statistical analyses.

## Results

### Circulatory T cell immune activity is heightened after cardiac arrest and resuscitation

**Figure 2:**
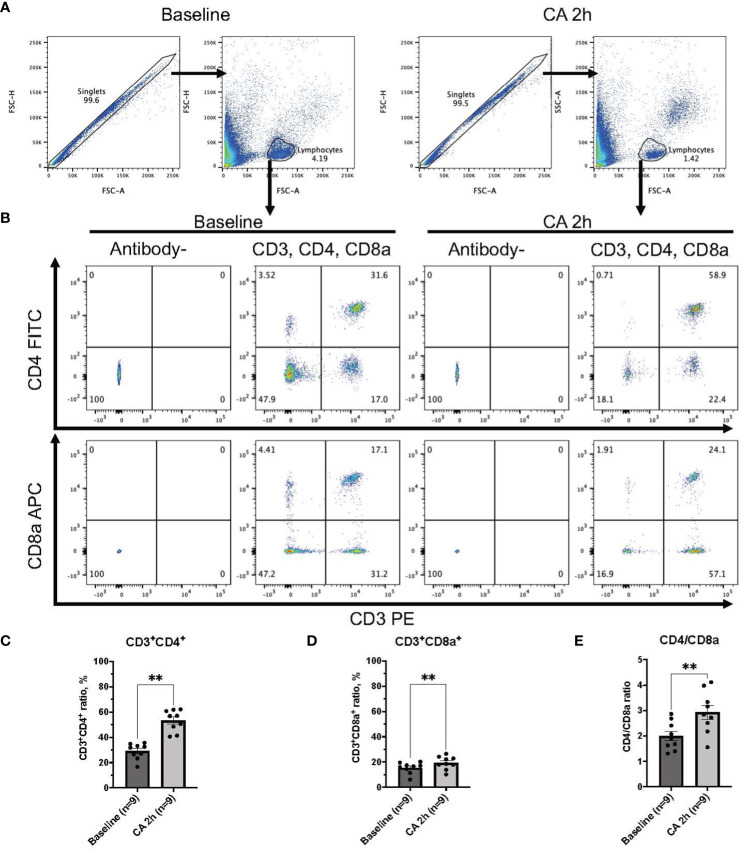
CD3, CD4, and CD8a expression in whole blood of rat CA model. **(A)** Representative images of dot plots through gating process. Lymphocytes were gated. **(B)** Representative dot plot images of CD3, CD4, and CD8a positive leukocytes. **(C)** CD3^+^CD4^+^ ratio in lymphocytes population. **(D)** CD3^+^CD8a^+^ ratio in lymphocytes population. **(E)** CD4/CD8a ratio. Data were expressed as means ± SEM (n = 9 rats/group). Two groups were compared by Wilcoxon matched-pairs signed rank test (** *P* < 0.01).

**Figure 3:**
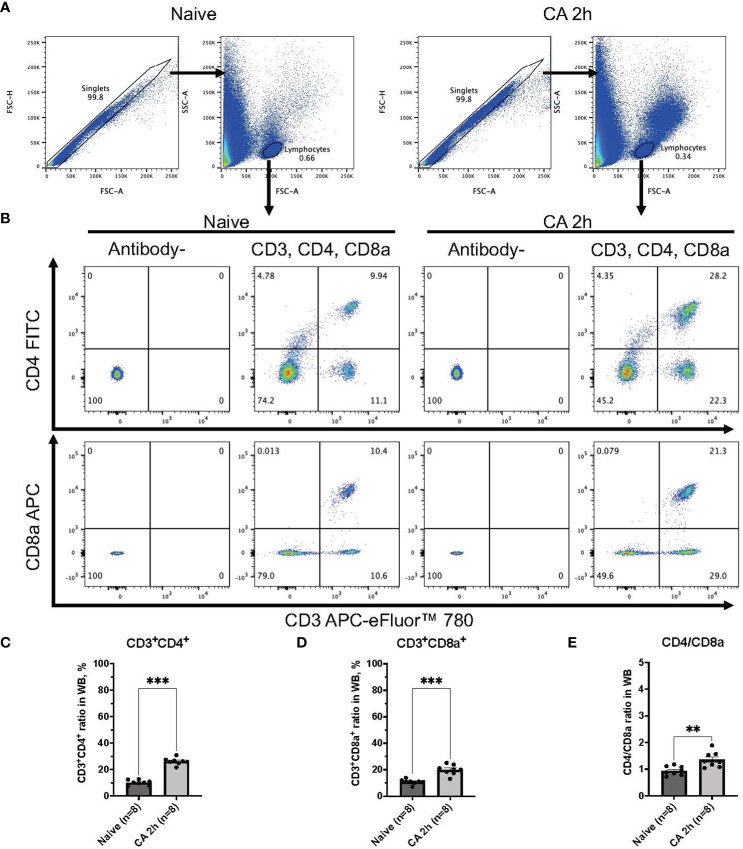
CD3, CD4, and CD8a expression in whole blood of mouse CA model. **(A)** Representative images of dot plots through gating process. Lymphocytes were gated. **(B)** Representative dot plot images of CD3, CD4, and CD8a positive leukocytes. **(C)** CD3^+^CD4^+^ ratio in lymphocytes population. **(D)** CD3^+^CD8a^+^ ratio in lymphocytes population. **(E)** CD4/CD8a ratio. Data were expressed as means ± SEM (n = 8 mice/group). Two groups were compared by Mann-Whitney test (** *P* < 0.01, *** *P* < 0.001).

**Figure 4:**
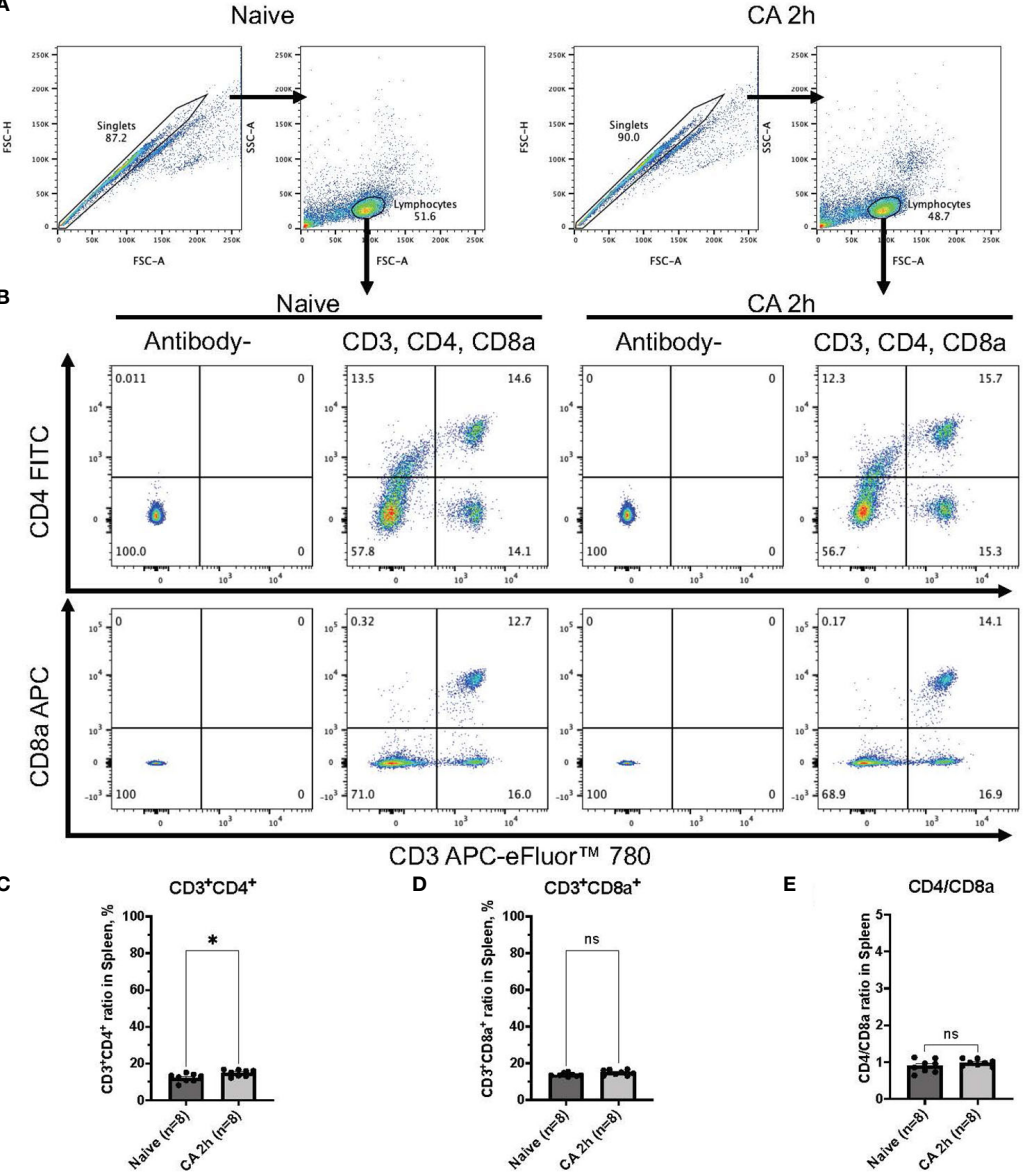
CD3, CD4, and CD8a expression in the spleen of mouse CA model. **(A)** Representative images of dot plots through gating process. Lymphocytes were gated. **(B)** Representative dot plot images of CD3, CD4, and CD8a positive leukocytes. **(C)** CD3^+^CD4^+^ ratio in lymphocytes population. **(D)** CD3^+^CD8a^+^ ratio in lymphocytes population. **(E)** CD4/CD8a ratio. Data were expressed as means ± SEM (n = 8 mice/group). Two groups were compared by Mann-Whitney test (* *P* < 0.05).

In a rat model of CA, ROSC was achieved at 58.4 ± 4.4 seconds (**Table 1**). At 2 hours after resuscitating from 12 minutes of asphyxia CA, whole blood samples were collected for subsequent flow cytometry analyses. The population of whole lymphocytes was gated on forward scatter/side scatter dot plots, and specific populations of immune cells, including CD3^+^CD4^+^ helper T lymphocytes and CD3^+^CD8a^+^ cytotoxic T lymphocytes, were identified (**Figures 2A, B**). In comparison to the baseline, CD3^+^CD4^+^ helper T lymphocytes markedly increased (**Figure 2C**, baseline, 29.02 ± 2.08 %; CA 2h, 53.27 ± 2.80 %, *P* < 0.01), while CD3^+^CD8a^+^ cytotoxic T lymphocytes increased slightly (**Figure 2D**, baseline, 15.42 ± 1.45 %; CA 2h, 19.34 ± 1.72 %, *P* < 0.01), consequently resulting in an increase of CD4/CD8a ratio (**Figure 2E**, baseline, 2.00 ± 0.19; CA 2h, 2.93 ± 0.27, *P* < 0.01). Similarly, in a mouse model of CA, ROSC was achieved at 81.3 ± 6.0 seconds (**Table 1**). At 2 hours after resuscitating from 8 minutes of KCl-induced CA, whole blood and spleen samples were collected. As depicted in **Figures 3A, B**, CD3^+^CD4^+^ helper T lymphocytes in whole blood displayed a notable increase (**Figure 3C**, Naïve, 10.05 ± 0.68 %; CA 2h, 26.16 ± 0.92 %, *P* < 0.001), while CD3^+^CD8a^+^ cytotoxic T lymphocytes increased (**Figure 3D**, Naïve, 10.82 ± 0.66 %; CA 2h, 19.91 ± 1.33 %, *P* < 0.001), ultimately leading to an increase in the CD4/CD8a ratio (**Figure 3E**, Naïve, 0.94 ± 0.06; CA 2h, 1.36 ± 0.10, *P* < 0.01). Although CD3^+^CD4^+^ helper T lymphocytes in the spleen also significantly increased (**Figures 4A–C**, Naïve, 12.17 ± 0.78 %; CA 2h, 14.83 ± 0.63 %, *P* < 0.05), CD3^+^CD8a^+^ cytotoxic T lymphocytes (**Figure 4D**, Naïve, 13.71 ± 0.32 %; CA 2h, 14.93 ± 0.52 %, *P* = 0.12) and the CD4/CD8a ratio did not exhibit significant increases (**Figure 4E**, Naïve, 0.90 ± 0.06; CA 2h, 0.99 ± 0.03, *P* = 0.31). These findings collectively suggest that there is an upregulation of circulatory immune activity, predominantly driven by an increased population of CD4^+^ T lymphocytes after CA.

**Table 1:** Baseline characteristics of the animals.

Rat			
Variable	CA 2h n=9		
Body weight, g	454.9 ± 9.7		
Before CA			
MAP, mmHg	89.6 ± 4.3		
HR, bpm	299.4 ± 11.1		
RR,/min	45.0 ± 0.1		
BT, °C	36.7 ± 0.2		
Time to ROSC, sec	58.4 ± 4.4		
Mouse			
Variable	Naïve n=8	CA 2h n=8	CA 24h n=3
Body weight, g	26.1 ± 0.6	26.9 ± 0.5	29.7 ± 0.3
Before CA			
MAP, mmHg	-	74.2 ± 3.9	70.5 ± 3.9
HR, bpm	-	542.5 ± 14.9	519.3 ± 22.6
RR,/min	-	110.1 ± 0.4	110.3 ± 0.7
BT, °C	-	36.8 ± 0.1	36.7 ± 0.1
Time to ROSC, sec	-	81.3 ± 6.0	80.7 ± 8.2
24h Survival rate	-	-	100% (3/3)

Data are presented as mean ± standard error.

CA, cardiac arrest; MAP, mean arterial pressure; HR, heart rate; RR, respiratory rate; BT, body temperature; ROSC, return of spontaneous circulation.

### Both nucleotide-metabolizing enzymes, CD39 and CD73, are increased promptly in the circulatory blood and lately in the spleen after cardiac arrest and resuscitation

**Figure 5:**
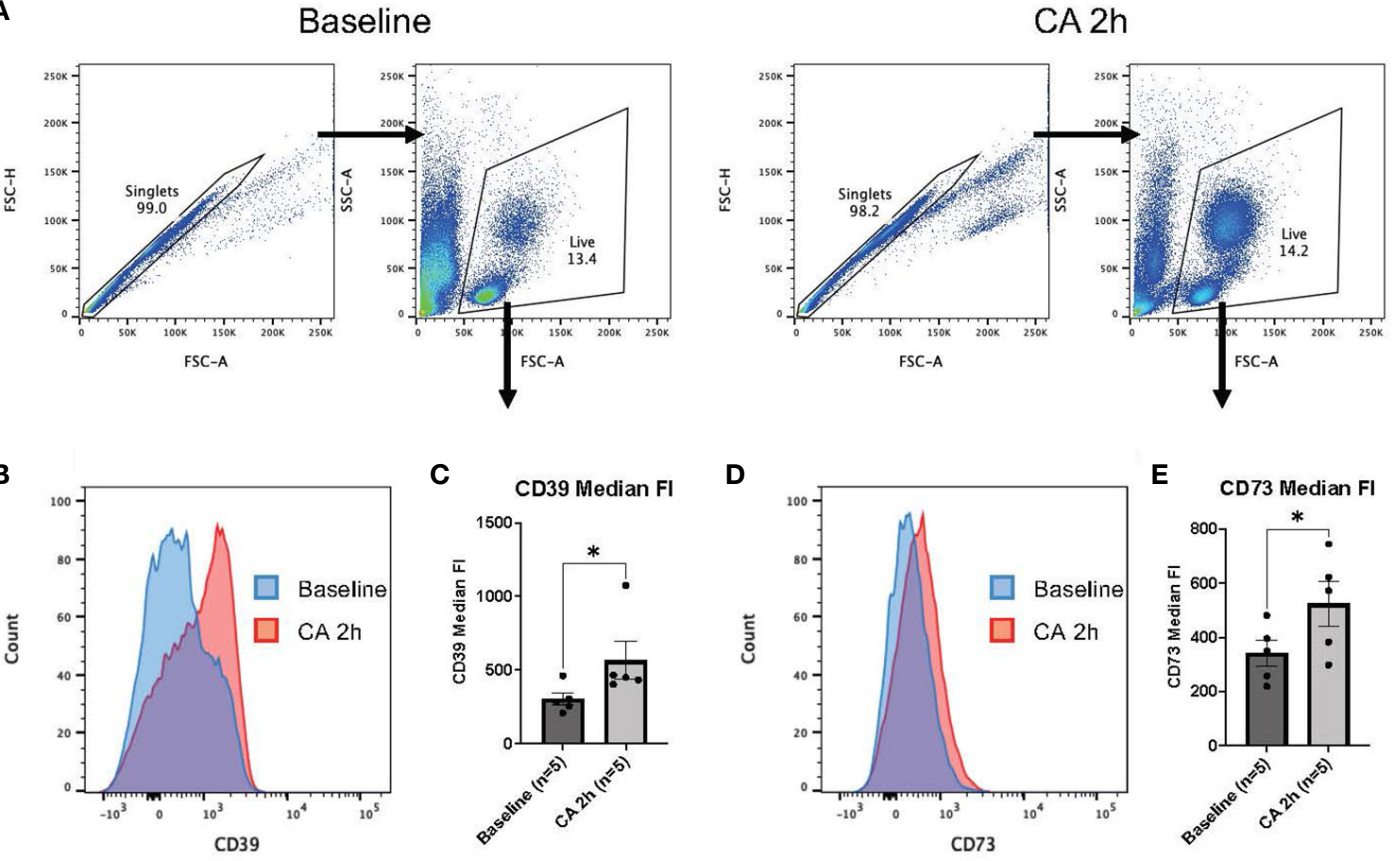
CD39 and CD73 expression in whole blood of rat CA model. **(A)** Representative images of dot plots through gating process. Leukocytes were gated. **(B)** Representative overlaid histograms of the CD39 fluorescence intensity. **(C)** CD39 median fluorescence intensity. **(D)** Representative overlaid histograms of CD73 fluorescence intensity. **(E)** CD73 median fluorescence intensity. Data were expressed as means ± SEM (n = 5 rats/group). Two groups were compared by Wilcoxon matched-pairs signed rank test (* *P* < 0.05).

**Figure 6:**
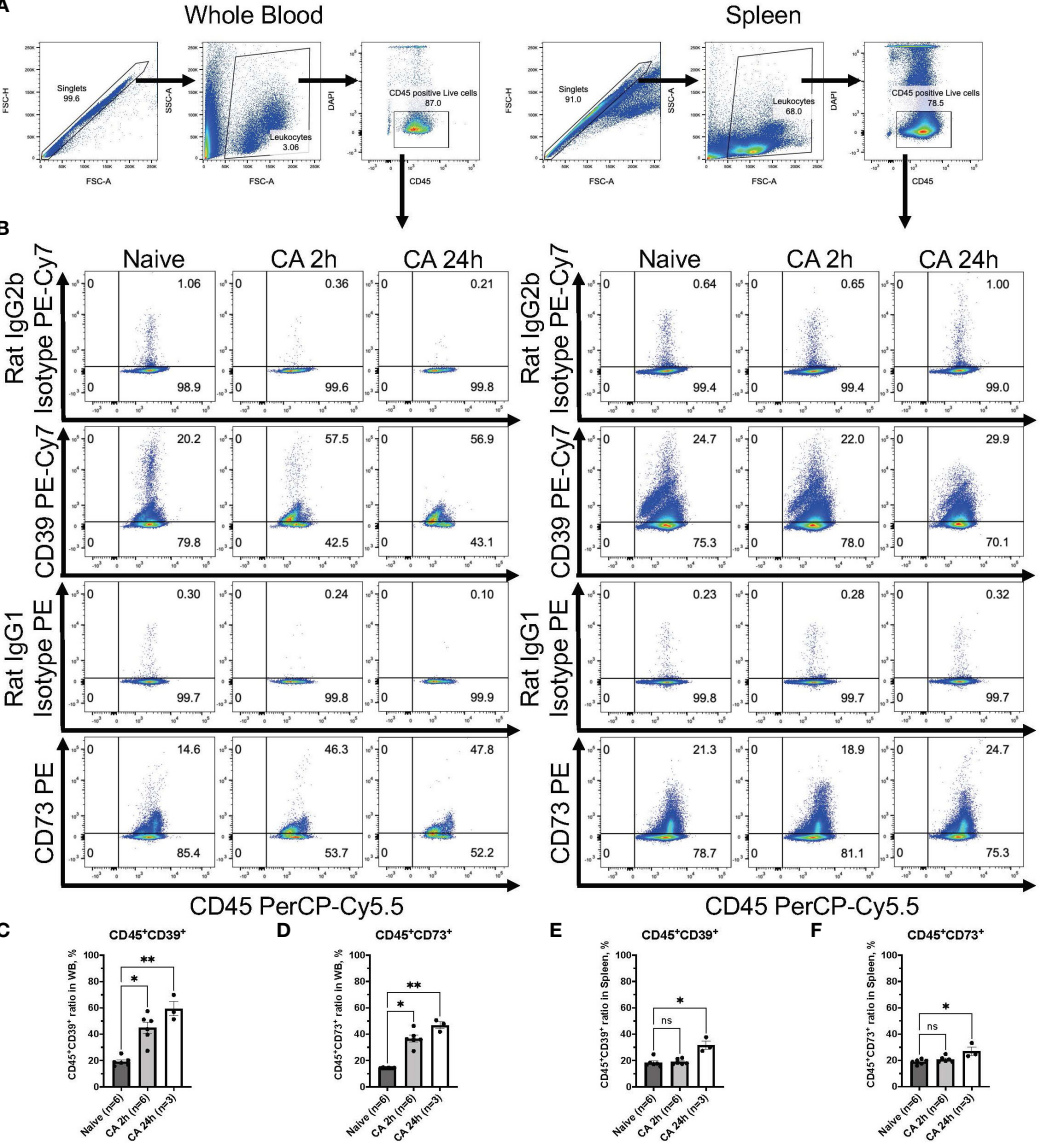
CD39 and CD73 expression in CD45^+^ leukocytes from whole blood and the spleen of mouse CA model. **(A)** Representative images of dot plots through gating process. CD45^+^DAPI^-^ leukocytes were gated. **(B)** Representative dot plot images of CD39, CD73, isotype controls, and CD45. **(C)** CD45^+^CD39^+^ ratio and **(D)** CD45^+^CD73^+^ ratio in CD45^+^ leukocytes in whole blood. **(E)** CD45^+^CD39^+^ ratio and **(F)** CD45^+^CD73^+^ ratio in CD45^+^ leukocytes in the spleen. Data were expressed as means ± SEM (n = 3-6 mice/group). Between naïve and 2 hours or 24 hours after CA groups, two groups were compared by Dunn’s multiple comparisons test (* *P* < 0.05, ** *P* < 0.01).

To further investigate immune responses after CA, our study focused on ectonucleotidases, specifically CD39 and CD73, known for their involvement in promoting immunosuppression through purinergic signaling and the metabolism of eATP (21–23). In a rat model of CA, median fluorescent intensity (FI) of both CD39 and CD73 were measured among leukocytes isolated from the whole blood (**Figures 5A–E**). Manually fluorophore-conjugated polyclonal antibodies targeting CD39 and CD73 detected a significant increase in the median FI of both antibodies 2 hours after CA (**Figures 5C, E**, CD39: baseline, 301.88 ± 42.94; CA 2h, 564.51 ± 127.80, *P* < 0.05 and CD73: baseline, 341.32 ± 47.03; CA 2h, 524.35 ± 81.23, *P* < 0.05). For a more detailed analysis, the expression of both ectonucleotidases were evaluated in a mouse model of CA. As shown in **Figures 6A, B**, CD45^+^ live leukocytes were gated in the whole blood and spleen, and positive ratios of both CD39 and CD73 were determined. In the whole blood, CD45^+^CD39^+^ leukocytes markedly increased (**Figure 6C**, Naïve, 19.18 ± 1.40 %; CA 2h, 44.90 ± 4.32 %, *P* < 0.05; CA 24h, 59.40 ± 5.48 %, *P* < 0.01), as did CD45^+^CD73^+^ leukocytes (**Figure 6D**, Naïve, 14.40 ± 0.12 %; CA 2h, 36.70 ± 2.50 %, *P* < 0.05; CA 24h, 46.77 ± 2.56 %, *P* < 0.01). However, in the spleen, both CD45^+^CD39^+^ (**Figure 6E**, Naïve, 18.35 ± 1.32 %; CA 2h, 18.98 ± 0.86 %, *P* = 0.88; CA 24h, 31.63 ± 3.12 %, *P* < 0.05) and CD45^+^CD73^+^ leukocytes (**Figure 6F**, Naïve, 18.88 ± 0.76 %; CA 2h, 20.75 ± 0.92 %, *P* = 0.49; CA 24h, 27.07 ± 3.09 %, *P* < 0.05) had increases at 24 hours but not at 2 hours after CA. These findings collectively indicated prompt ectonucleotidase increases in the circulatory blood and late increases in the spleen after CA.

### Myeloid cells had markedly high levels of CD39 and CD73 in both the circulatory blood and the spleen after cardiac arrest and resuscitation

**Figure 7:**
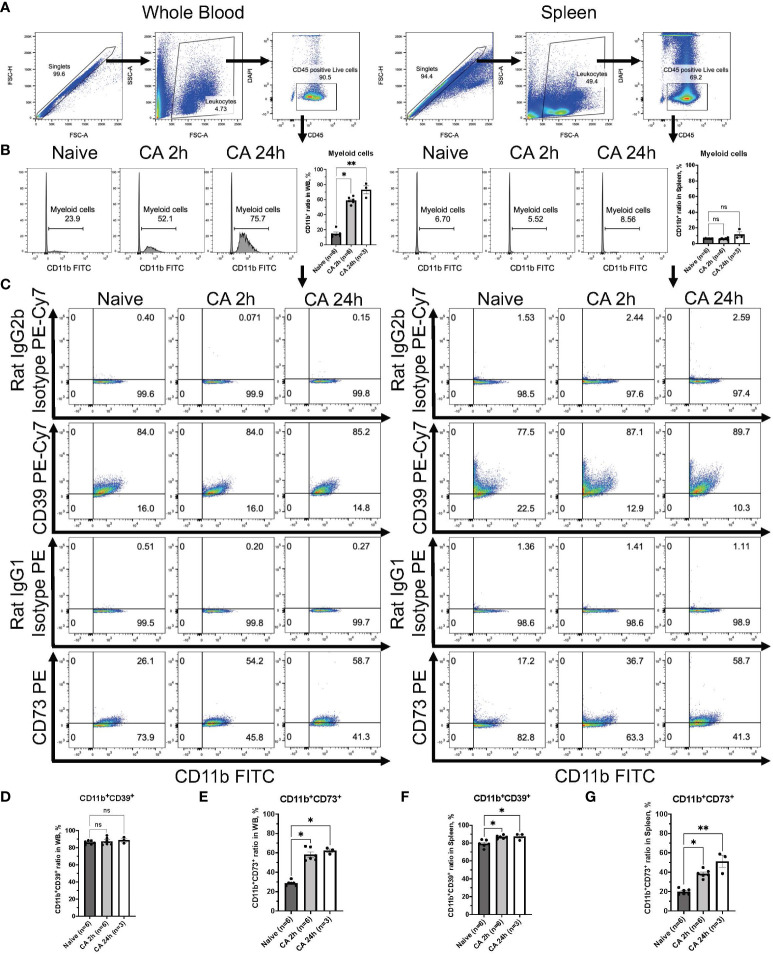
CD39 and CD73 expression in CD11b^+^ myeloid cells from whole blood and the spleen of mouse CA model. **(A)** Representative images of dot plots through gating process. CD45^+^DAPI^-^ leukocytes were gated. **(B)** Representative histogram images of CD11b in CD45^+^ leukocytes. CD11b^+^ myeloid cells were gated. CD11b^+^ ratio in CD45^+^ leukocytes. **(C)** Representative dot plot images of CD39, CD73, isotype controls, and CD11b. **(D)** CD11b^+^CD39^+^ ratio and **(E)** CD11b^+^CD73^+^ ratio in CD11b^+^ myeloid cells in whole blood. **(F)** CD11b^+^CD39^+^ ratio and **(G)** CD11b^+^CD73^+^ ratio in CD11b^+^ myeloid cells in the spleen. Data were expressed as means ± SEM (n = 3-6 mice/group). Between naïve and 2 hours or 24 hours after CA groups, two groups were compared by Dunn’s multiple comparisons test (* *P* < 0.05, ** *P* < 0.01).

To elucidate the specific immune cell populations in the whole blood and the spleen that can increase ectonucleotidases on the cell surface after CA, we conducted further analyses focusing on CD11b^+^ myeloid cells, CD3^+^ T lymphocytes, and CD19^+^ B lymphocytes by using the mouse model. Following gating of CD45^+^DAPI^-^ leukocytes (**Figure 7A**), CD11b^+^ myeloid cells were gated, showing the remarkable increase of myeloid cells in the circulatory blood (**Figure 7B**, Naïve, 14.70 ± 1.94 %; CA 2h, 58.95 ± 1.83 %, *P* < 0.05; CA 24h, 73.03 ± 5.59 %, *P* < 0.01). The expression of CD39 or CD73 in myeloid cells was graphically represented and analyzed (**Figure 7C**). Notably, myeloid cells essentially had high levels of CD39, which was retained until 24 hours after CA (**Figure 7D**, Naïve, 86.22 ± 1.10 %; CA 2h, 87.58 ± 2.06 %, *P* > 0.99; CA 24h, 88.80 ± 2.05 %, *P* = 0.43), while, the expression of CD73 markedly increased in the whole blood (**Figure 7E**, Naïve, 28.63 ± 1.07 %; CA 2h, 58.17 ± 2.71, *P* < 0.05; CA 24h, 62.17 ± 1.81, *P* < 0.05). Similarly, in the spleen, the expression of both CD39 and CD73 in myeloid cells were shown to increase (**Figures 7F, G**, CD11b^+^CD39^+^: Naïve, 79.43 ± 1.68 %; CA 2h, 86.90 ± 0.76 %, *P* < 0.05; CA 24h, 87.53 ± 2.22 %, *P* < 0.05 and CD11b^+^CD73^+^: Naïve, 19.85 ± 1.21 %; CA 2h, 38.47 ± 1.71, *P* < 0.05; CA 24h, 51.10 ± 6.30 %, *P* < 0.01).

### Increases of both CD39 and CD73 were observed in the circulatory B lymphocytes but not in the T lymphocytes

**Figure 8:**
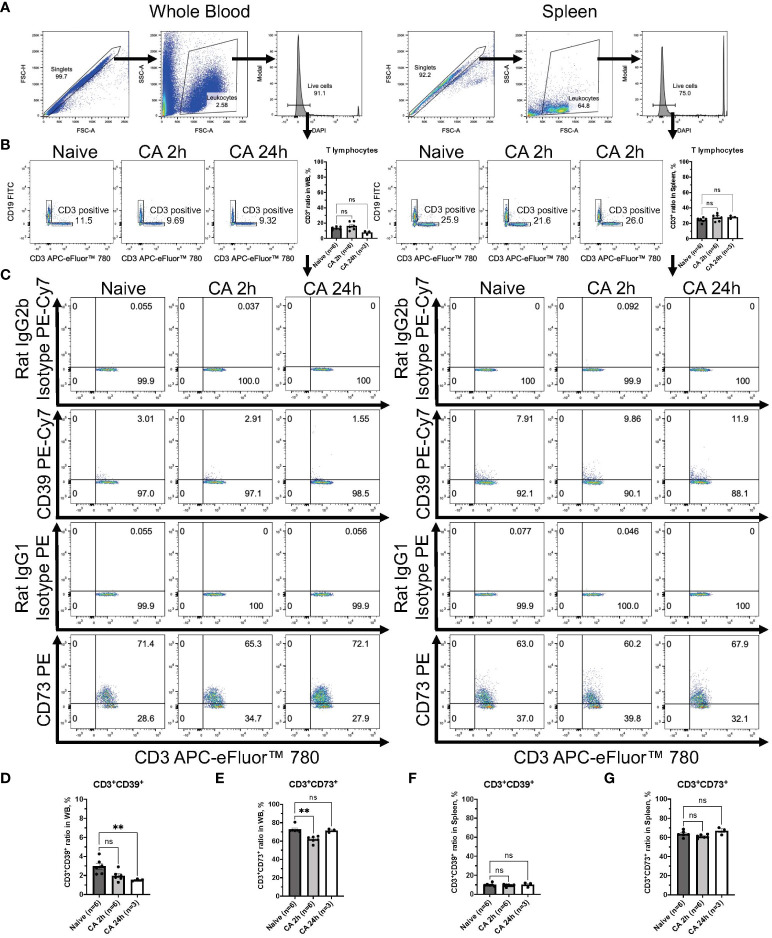
CD39 and CD73 expression in CD3^+^ T lymphocytes from whole blood and the spleen of mouse CA model. **(A)** Representative images of dot plots through gating process. DAPI^-^ leukocytes were gated. **(B)** Representative dot plot images of CD19 and CD3 in leukocytes. CD3^+^ T lymphocytes were gated. CD3^+^ ratio in leukocytes. **(C)** Representative dot plot images of CD39, CD73, isotype controls, and CD3. **(D)** CD3^+^CD39^+^ ratio and **(E)** CD3^+^CD73^+^ ratio in CD3^+^ T lymphocytes in whole blood. **(F)** CD3^+^CD39^+^ ratio and **(G)** CD3^+^CD73^+^ ratio in CD3^+^ T lymphocytes in the spleen. Data were expressed as means ± SEM (n = 3-6 mice/group). Between naïve and 2 hours or 24 hours after CA groups, two groups were compared by Dunn’s multiple comparisons test (** *P* < 0.01).

**Figure 9:**
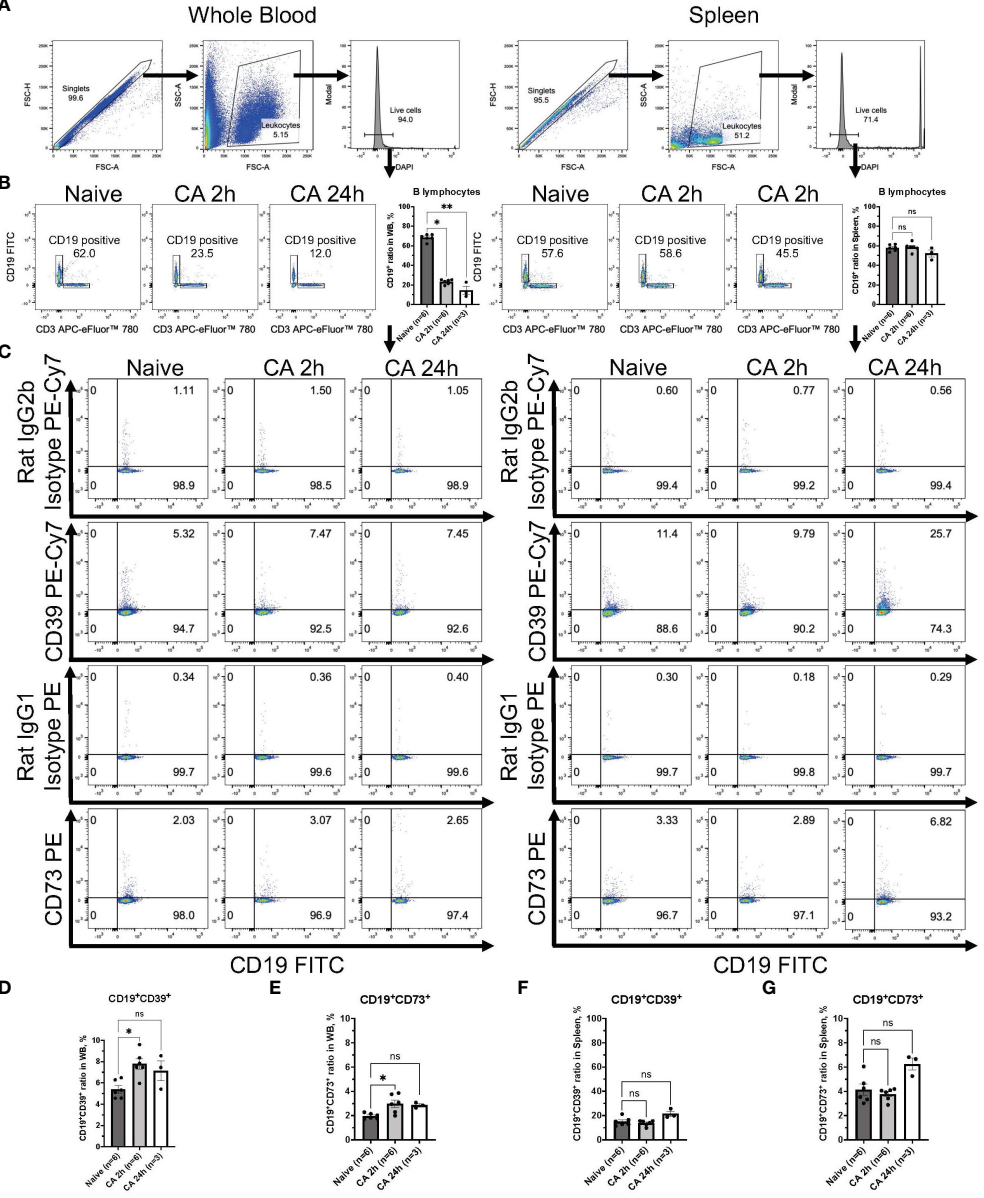
CD39 and CD73 expression in CD19^+^ B lymphocytes from whole blood and the spleen of mouse CA model. **(A)** Representative images of dot plots through gating process. DAPI^-^ leukocytes were gated. **(B)** Representative dot plot images of CD19 and CD3 in leukocytes. CD19^+^ B lymphocytes were gated. CD19^+^ ratio in leukocytes. **(C)** Representative dot plot images of CD39, CD73, isotype controls, and CD19. **(D)** CD19^+^CD39^+^ ratio and **(E)** CD19^+^CD73^+^ ratio in CD19^+^ B lymphocytes in whole blood. **(F)** CD19^+^CD39^+^ ratio and **(G)** CD19^+^CD73^+^ ratio in CD19^+^ B lymphocytes in the spleen. Data were expressed as means ± SEM (n = 3-6 mice/group). Between naïve and 2 hours or 24 hours after CA groups, two groups were compared by Dunn’s multiple comparisons test (* *P* < 0.05, ** *P* < 0.01).

Regarding the expression of ectonucleotidases within lymphocytes, following gating of CD3^+^ T lymphocytes or CD19^+^ B lymphocytes in DAPI^-^ live leukocytes population (**Figures 8A, B** and **9A, B**), the expression of these enzymes was evaluated in both CD3^+^ T lymphocytes and CD19^+^ B lymphocytes (**Figures 8C**, **9C**). The population of CD19^+^ B lymphocytes remarkably decreased in the whole blood (**Figure 9B**, Naïve, 68.37 ± 1.43 %; CA 2h, 23.22 ± 0.99 %, *P* < 0.05; CA 24h, 14.40 ± 4.33, *P* < 0.01). Both CD3^+^CD39^+^ and CD3^+^CD73^+^ T lymphocytes had decreases in the whole blood (**Figures 8D, E**, CD3^+^CD39^+^: Naïve, 2.97 ± 0.33 %; CA 2h, 1.97 ± 0.22 %, *P* = 0.07; CA 24h, 1.53 ± 0.07, *P* < 0.01 and CD3^+^CD73^+^: Naïve, 72.83 ± 1.57 %; CA 2h, 61.88 ± 1.46, *P* < 0.01; CA 24h, 71.53 ± 1.11, *P* > 0.99). In contrast, both CD19^+^CD39^+^ and CD19^+^CD73^+^ B lymphocytes were shown to have significant increases (**Figures 9D, E**, CD19^+^CD39^+^: Naïve, 5.39 ± 0.34 %; CA 2h, 7.78 ± 0.49 %, *P* < 0.05; CA 24h, 7.14 ± 0.92, *P* = 0.18 and CD19^+^CD73^+^: Naïve, 1.96 ± 0.09 %; CA 2h, 2.99 ± 0.31, *P* < 0.05; CA 24h, 2.86 ± 0.12, *P* = 0.07) in the whole blood. In the spleen, no marked differences were evident in either CD3^+^ T lymphocytes (**Figures 8F, G**, CD3^+^CD39^+^: Naïve, 10.06 ± 0.70 %; CA 2h, 9.39 ± 0.52 %, *P* > 0.99; CA 24h, 10.21 ± 1.40, *P* > 0.99 and CD3^+^CD73^+^: Naïve, 63.70 ± 1.33 %; CA 2h, 61.02 ± 0.76, *P* = 0.27; CA 24h, 66.90 ± 2.25, *P* = 0.95) or CD19^+^ B lymphocytes (**Figures 9F, G**, CD19^+^CD39^+^: Naïve, 15.00 ± 1.45 %; CA 2h, 13.77 ± 0.91 %, *P* > 0.99; CA 24h, 21.43 ± 2.15, *P* = 0.11 and CD19^+^CD73^+^: Naïve, 4.14 ± 0.46 %; CA 2h, 3.77 ± 0.21, *P* > 0.99; CA 24h, 6.25 ± 0.50, *P* = 0.09). These findings provide insight into the immune cell populations that are predisposed to ectonucleotidase expression, with a distinct upregulation observed in myeloid cells in the blood circulation and the spleen after CA.

## Discussion

The present study comprehensively explored the expression levels of ectonucleotidases. This investigation represents the inaugural exploration of CD39 and CD73 expression within distinct immune cell populations in our rodent model of CA. It is noteworthy that, while our prior research has demonstrated upregulation of RNA levels of *Cd39* and *Cd73* in the brain and heart at 2 hours after CA and resuscitation, the expression of these ectonucleotidases on the cellular surface of circulating immune cells has not yet been addressed (19).

Ryzhov and colleagues conducted an important study in which CD39 and CD73 levels in leukocytes were assessed in patients after CA at distinct times over one week after resuscitation. Their findings indicated that CD39 and CD73 expression rapidly increased, peaking at 6 and 12 hours after resuscitation and persisting (24). No B cell alterations were noted. Nevertheless, it is important to acknowledge that drawing comprehensive mechanistic conclusions from their study is challenging due to the relatively small sample size, missing data points, and heterogeneity of patient characteristics, some of whom underwent therapeutic temperature management. More importantly, there has been no data elucidating which population of leukocytes is predominantly involved in purinergic signaling. Our data provides experimental evidence support for the substantive increases of CD39 and CD73 on circulating immune cell populations following CA and in PCAS.

In separate research studies unrelated to CA and resuscitation, Köhler and colleagues demonstrated the protective effects of CD39 in the context of IRI using mouse models of myocardial infarction. Here, the preconditioning therapy enhanced CD39 expression in cardiomyocytes and endothelial cells within 2 hours of initiating the therapy, resulting in a significant reduction in infarct size in the heart. Notably, the protective effects were compromised in mice with *Cd39* gene deletion, highlighting the crucial role of CD39 in this process (25). In the peripheral blood, CD39 is constitutively expressed in over 90% of B cells, over 90% of monocytes, and approximately 20-30% of CD4^+^ T cells (26). CD39 expression can be induced by various factors, including exposure to eATP, pro-inflammatory cytokines such as interleukin-6 (IL-6), as well as conditions of oxidative stress and hypoxia (17). Within the rodent population, CD11b^+^ leukocytes represent myeloid cells, including monocytes, macrophages, and granulocytes. Purinergic signaling has a notable impact on monocyte functions, as eATP not only serves as a chemoattractant for monocytes but also activates the NF-kB signaling pathway, promoting polarization towards M1 macrophages (17, 27). Furthermore, adenosine signaling via A2A and A2B receptors has the capacity to induce anti-inflammatory M2 macrophages. These M2 macrophages are characterized by a reduction in tumor necrosis factor-alpha (TNF-α) levels and an increase in anti-inflammatory IL-10 levels (28–30).

The monocyte-macrophage phagocytic system stands as a principal driver of inflammation in the context of IRI (31–35). Our published (36) and unpublished data have demonstrated a notable surge in proinflammatory cytokine levels, both within the affected tissues and in systemic circulation, within the initial 6 hours following resuscitation. However, an intriguing question arises concerning whether the integrity of the blood-brain barrier undergoes a transient disruption after CA, potentially facilitating the infiltration of circulating immune cells into the brain parenchyma. Notably, a study by Zhang and colleagues revealed the presence of various immune cell populations, including CD11b^+^CD11c^+^ dendritic cells, CD11b^+^CD45^+hi^ macrophages, CD11b^+^Ly6G^-^ monocytes, and cytotoxic CD8^+^ T cells, in brain parenchyma 3 days after resuscitation. This coincided with increased numbers of these cell populations observed in the blood and bone marrow (37). Collectively, these findings emphasize the pivotal role of recruited immune cells, particularly bone-marrow-derived circulating monocytes and macrophages, in orchestrating the post-CA immune response within the brain. Notably, our current study provides additional insights by revealing a significant upregulation of ectonucleotidase expression within the circulatory CD11b^+^ myeloid cells, concomitant with a substantial increase in their population. This populational shift of myeloid cells likely constitutes a pivotal factor in the increased levels of ectonucleotidases observed in whole leukocytes. Of particular interest is the remarkable upregulation of CD73, indicating that myeloid cells, which encompass monocytes and macrophages, are likely to play a prominent role in immune responses and purinergic signaling following CA and resuscitation.

In the context of global cerebral ischemia caused by CA and resuscitation, the traditionally immune-privileged brain undergoes rapid infiltration of pro-inflammatory T cells (38). Predominantly, these infiltrating cells are CD4^+^ T lymphocytes, which not only become activated but also maintain their presence within the cerebral parenchyma for an extended duration, persisting up to 72 hours after resuscitation (39, 40). In experimental stroke studies, the timing of immune cell entry into the brain has been well-documented, with neutrophils being the initial responders, appearing within a few hours of stroke onset, followed by lymphocytes, which typically arrive 12 to 24 hours after the stroke (39–42). This lymphocytic infiltration coincides with a significant increase in neuronal cell death in the hippocampal CA1 region, a phenomenon possibly mediated by the secretion of pro-inflammatory cytokines like TNFα and IFNγ (41, 43). These observations suggest that modulating T cell infiltration and the subsequent immune cascade in the aftermath of CA could represent a novel therapeutic approach (44). Our current findings from both rodent models also reveal that it is CD3^+^CD4^+^ helper T lymphocytes, rather than CD3^+^CD8a^+^ cytotoxic T lymphocytes, that undergo upregulation, leading to an increased CD4/CD8a ratio within the circulatory system following CA. These results collectively indicate an elevation in circulatory immune activity, primarily driven by an expanded population of CD4^+^ T lymphocytes after CA. Intriguingly, CD39 and CD73 exhibit upregulation within the B lymphocyte population, while their expression decreases in the T lymphocyte population. These findings shed light on the specific immune cell populations prone to ectonucleotidase expression, with a distinct increase evident in myeloid cells and B lymphocytes after CA.

This study presents a number of limitations. Firstly, our approach utilized cytometric analyses to assess alterations in predominant immune cell populations and the expression of ectonucleotidase following CA. While this method provides valuable insights, the generalizability of our findings as applied to human condition of PCAS may be constrained. Fundamental responses in rodents may not mimic human inflammatory diseases (45). However, the upregulation of purinergic signaling represented by increases of CD39/CD73 found in both humans (24) and our rodent models strongly support the need for further mechanistic studies.

The most applicable and relevant animal model of PCAS has been controversial for decades (46). Of the available models, rodents have the advantage of commercially available bioassays and share many physiological similarities with humans. It is worth noting that we employed both rat and mouse models of CA in the current study, revealing analogous shifts in the population of CD3^+^CD4^+^ helper T lymphocytes and CD3^+^CD8a^+^ cytotoxic T lymphocytes after CA across two different species. Due to the limited availability on conjugated antibodies targeting immune cell subpopulations for rats, it was imperative for us to utilize mice in order to obtain the detailed information on ectonucleotidase expressions after CA. Our evaluation was further based on the analysis of whole blood and spleen samples collected at two time points, specifically 2 and 24 hours after CA. Consequently, any potential variations in the immune cell profile occurring at time points other than 2 or 24 hours remain unexplored. We may have failed to detect significant changes that occur at other time points. Therefore, further investigations into the dynamic changes in immune cell profiles over various time intervals are warranted.

In conclusion, our study has revealed an upregulation in circulatory immune activity, primarily attributed to the expanded population of CD4^+^ T lymphocytes. Furthermore, our findings have elucidated the immune cell populations that are likely to express CD39 and CD73, with notably increased levels observed in blood myeloid cells and B lymphocytes, following CA. These findings provide a foundation for future studies to explore the therapeutic potential of modulating immune responses and purinergic signaling following CA and resuscitation.

## Data availability statement

The original contributions presented in the study are included in the article/supplementary material. Further inquiries can be directed to the corresponding author.

## Ethics statement

The animal study was approved by The Institutional Animal Care and Use Committees of Feinstein Institutes for Medical Research. The study was conducted in accordance with the local legislation and institutional requirements.

## Author contributions

TA: Data curation, Formal analysis, Investigation, Methodology, Project administration, Validation, Visualization, Writing – original draft, Writing – review & editing. VW: Investigation, Writing – review & editing. TY: Investigation, Writing – review & editing. EN: Writing – review & editing. YE: Writing – review & editing. KH: Writing – review & editing. SR: Writing – review & editing. HN: Writing – review & editing. BD: Writing – review & editing. SK: Methodology, Writing – review & editing. AM: Writing – review & editing. PW: Writing – review & editing. LB: Conceptualization, Funding acquisition, Writing – review & editing. KS: Conceptualization, Funding acquisition, Project administration, Supervision, Writing – review & editing.

## References

[1] Benjamin EJ Blaha MJ Chiuve SE Cushman M Das SR Deo R. Heart disease and stroke statistics-2017 update: A report from the American Heart Association. Circulation. (2017) 135:e146–603. doi: 10.1161/CIR.0000000000000485 PMC5408160 28122885

[2] Nolan JP Neumar RW Adrie C Aibiki M Berg RA Bottiger BW. Post-cardiac arrest syndrome: epidemiology, pathophysiology, treatment, and prognostication. A Scientific Statement from the International Liaison Committee on Resuscitation; the American Heart Association Emergency Cardiovascular Care Committee; the Council on Cardiovascular Surgery and Anesthesia; the Council on Cardiopulmonary, Perioperative, and Critical Care; the Council on Clinical Cardiology; the Council on Stroke. Resuscitation. (2008) 79:350–79. doi: 10.1016/j.resuscitation.2008.09.017 18963350

[3] Callaway CW Donnino MW Fink EL Geocadin RG Golan E Kern KB. Part 8: post-Cardiac arrest care: 2015 American Heart Association guidelines update for cardiopulmonary resuscitation and emergency cardiovascular care. Circulation. (2015) 132:S465–82. doi: 10.1161/CIR.0000000000000262 PMC4959439 26472996

[4] Aoki T Wong V Endo Y Hayashida K Takegawa R Okuma Y. Bio-physiological susceptibility of the brain, heart, and lungs to systemic ischemia reperfusion and hyperoxia-induced injury in post-cardiac arrest rats. Sci Rep. (2023) 13:3419. doi: 10.1038/s41598-023-30120-1 36854715 PMC9974929

[5] Cunningham CA Coppler PJ Skolnik AB. The immunology of the post-cardiac arrest syndrome. Resuscitation. (2022) 179:116–23. doi: 10.1016/j.resuscitation.2022.08.013 36028143

[6] Grimaldi D Sauneuf B Guivarch E Ricome S Geri G Charpentier J. High level of endotoxemia following out-of-hospital cardiac arrest is associated with severity and duration of postcardiac arrest shock. Crit Care Med. (2015) 43:2597–604. doi: 10.1097/CCM.0000000000001303 26427593

[7] Peberdy MA Andersen LW Abbate A Thacker LR Gaieski D Abella BS. Inflammatory markers following resuscitation from out-of-hospital cardiac arrest-A prospective multicenter observational study. Resuscitation. (2016) 103:117–24. doi: 10.1016/j.resuscitation.2016.01.006 26826561

[8] Adrie C Adib-Conquy M Laurent I Monchi M Vinsonneau C Fitting C. Successful cardiopulmonary resuscitation after cardiac arrest as a "sepsis-like" syndrome. Circulation. (2002) 106:562–8. doi: 10.1161/01.CIR.0000023891.80661.AD 12147537

[9] Beurskens CJ Horn J de Boer AM Schultz MJ van Leeuwen EM Vroom MB. Cardiac arrest patients have an impaired immune response, which is not influenced by induced hypothermia. Crit Care. (2014) 18:R162. doi: 10.1186/cc14002 25078879 PMC4261599

[10] Soppi E Lindroos M Nikoskelainen J Kalliomaki JL. Effect of cardiopulmonary resuscitation-induced stress on cell-mediated immunity. Intensive Care Med. (1984) 10:287–92. doi: 10.1007/BF00254317 6239889

[11] Hotchkiss RS Monneret G Payen D. Sepsis-induced immunosuppression: from cellular dysfunctions to immunotherapy. Nat Rev Immunol. (2013) 13:862–74. doi: 10.1038/nri3552 PMC4077177 24232462

[12] Chekol Abebe E Asmamaw Dejenie T Mengie Ayele T Dagnew Baye N Agegnehu Teshome A Tilahun Muche Z. The role of regulatory B cells in health and diseases: A systemic review. J Inflammation Res. (2021) 14:75–84. doi: 10.2147/JIR.S286426 PMC7811483 33469337

[13] Dou H Brandon NR Koper KE Xu Y. Fingerprint of circulating immunocytes as biomarkers for the prognosis of brain inflammation and neuronal injury after cardiac arrest. ACS Chem Neurosci. (2023) 14:4115–27. doi: 10.1021/acschemneuro.3c00397 PMC10704468 37967214

[14] Chadwick JW Fine N Khoury W Tasevski N Sun CX Boroumand P. Tissue-specific murine neutrophil activation states in health and inflammation. J Leukoc Biol. (2021) 110:187–95. doi: 10.1002/JLB.4AB1020-248RRR 33145850

[15] Eltzschig HK Eckle T. Ischemia and reperfusion–from mechanism to translation. Nat Med. (2011) 17:1391–401. doi: 10.1038/nm.2507 PMC3886192 22064429

[16] Eltzschig HK Sitkovsky MV Robson SC. Purinergic signaling during inflammation. N Engl J Med. (2013) 368:1260. doi: 10.1056/NEJMc1300259 23534573

[17] Allard B Longhi MS Robson SC Stagg J. The ectonucleotidases CD39 and CD73: Novel checkpoint inhibitor targets. Immunol Rev. (2017) 276:121–44. doi: 10.1111/imr.12528 PMC5338647 28258700

[18] Aoki T Wong V Endo Y Hayashida K Takegawa R Shoaib M. Insufficient oxygen inhalation during cardiopulmonary resuscitation induces early changes in hemodynamics followed by late and unfavorable systemic responses in post-cardiac arrest rats. FASEB J. (2023) 37:e23001. doi: 10.1096/fj.202202063R 37249913

[19] Shinozaki K Wong V Aoki T Hayashida K Takegawa R Endo Y. The role of pyruvate-induced enhancement of oxygen metabolism in extracellular purinergic signaling in the post-cardiac arrest rat model. Purinergic Signal. (2023). doi: 10.1007/s11302-023-09958-7 PMC11303634 37507639

[20] Han F Da T Riobo NA Becker LB. Early mitochondrial dysfunction in electron transfer activity and reactive oxygen species generation after cardiac arrest. Crit Care Med. (2008) 36:S447–53. doi: 10.1097/CCM.0b013e31818a8a51 PMC3315374 20449909

[21] Xia C Yin S To KKW Fu L. CD39/CD73/A2AR pathway and cancer immunotherapy. Mol Cancer. (2023) 22:44. doi: 10.1186/s12943-023-01733-x 36859386 PMC9979453

[22] Dong LW Ma ZC Fu J Huang BL Liu FJ Sun D. Upregulated adenosine 2A receptor accelerates post-infectious irritable bowel syndrome by promoting CD4+ T cells' T helper 17 polarization. World J Gastroenterol. (2022) 28:2955–67. doi: 10.3748/wjg.v28.i25.2955 PMC9280732 35978875

[23] Csoka B Himer L Selmeczy Z Vizi ES Pacher P Ledent C. Adenosine A2A receptor activation inhibits T helper 1 and T helper 2 cell development and effector function. FASEB J. (2008) 22:3491–9. doi: 10.1096/fj.08-107458 PMC2537430 18625677

[24] Ryzhov S May T Dziodzio J Emery IF Lucas FL Leclerc A. Number of circulating CD73-expressing lymphocytes correlates with survival after cardiac arrest. J Am Heart Assoc. (2019) 8:e010874. doi: 10.1161/JAHA.118.010874 31237169 PMC6662342

[25] Kohler D Eckle T Faigle M Grenz A Mittelbronn M Laucher S. CD39/ectonucleoside triphosphate diphosphohydrolase 1 provides myocardial protection during cardiac ischemia/reperfusion injury. Circulation. (2007) 116:1784–94. doi: 10.1161/CIRCULATIONAHA.107.690180 17909107

[26] Dwyer KM Deaglio S Gao W Friedman D Strom TB Robson SC. CD39 and control of cellular immune responses. Purinergic Signal. (2007) 3:171–80. doi: 10.1007/s11302-006-9050-y PMC2096766 18404431

[27] Levesque SA Kukulski F Enjyoji K Robson SC Sevigny J. NTPDase1 governs P2X7-dependent functions in murine macrophages. Eur J Immunol. (2010) 40:1473–85. doi: 10.1002/eji.200939741 PMC3045779 20201036

[28] Serra S Vaisitti T Audrito V Bologna C Buonincontri R Chen SS. Adenosine signaling mediates hypoxic responses in the chronic lymphocytic leukemia microenvironment. Blood Adv. (2016) 1:47–61. doi: 10.1182/bloodadvances.2016000984 29296695 PMC5744057

[29] Ferrante CJ Pinhal-Enfield G Elson G Cronstein BN Hasko G Outram S. The adenosine-dependent angiogenic switch of macrophages to an M2-like phenotype is independent of interleukin-4 receptor alpha (IL-4Ralpha) signaling. Inflammation. (2013) 36:921–31. doi: 10.1007/s10753-013-9621-3 PMC3710311 23504259

[30] Murphree LJ Sullivan GW Marshall MA Linden J. Lipopolysaccharide rapidly modifies adenosine receptor transcripts in murine and human macrophages: role of NF-kappaB in A(2A) adenosine receptor induction. Biochem J. (2005) 391:575–80. doi: 10.1042/BJ20050888 PMC1276958 16022683

[31] Tuttolomondo A Di Sciacca R Di Raimondo D Renda C Pinto A Licata G. Inflammation as a therapeutic target in acute ischemic stroke treatment. Curr Top Med Chem. (2009) 9:1240–60. doi: 10.2174/156802609789869619 19849665

[32] Jayaraj RL Azimullah S Beiram R Jalal FY Rosenberg GA. Neuroinflammation: friend and foe for ischemic stroke. J Neuroinflamm. (2019) 16:142. doi: 10.1186/s12974-019-1516-2 PMC6617684 31291966

[33] Mills EL Kelly B Logan A Costa ASH Varma M Bryant CE. Succinate dehydrogenase supports metabolic repurposing of mitochondria to drive inflammatory macrophages. Cell. (2016) 167:457–70.e13. doi: 10.1016/j.cell.2016.08.064 27667687 PMC5863951

[34] Fujiu K Shibata M Nakayama Y Ogata F Matsumoto S Noshita K. A heart-brain-kidney network controls adaptation to cardiac stress through tissue macrophage activation. Nat Med. (2017) 23:611–22. doi: 10.1038/nm.4326 28394333

[35] Ma Y Li Y Jiang L Wang L Jiang Z Wang Y. Macrophage depletion reduced brain injury following middle cerebral artery occlusion in mice. J Neuroinflamm. (2016) 13:38. doi: 10.1186/s12974-016-0504-z PMC4752808 26873581

[36] Shinozaki K Lampe JW Kim J Yin T Da T Oda S. The effects of early high-volume hemofiltration on prolonged cardiac arrest in rats with reperfusion by cardiopulmonary bypass: a randomized controlled animal study. Intensive Care Med Exp. (2016) 4:25. doi: 10.1186/s40635-016-0101-6 27612461 PMC5017966

[37] Zhang C Brandon NR Koper K Tang P Xu Y Dou H. Invasion of Peripheral Immune Cells into Brain Parenchyma after Cardiac Arrest and Resuscitation. Aging Dis. (2018) 9:412–25. doi: 10.14336/AD.2017.0926 PMC5988596 29896429

[38] Iadecola C Anrather J. The immunology of stroke: from mechanisms to translation. Nat Med. (2011) 17:796–808. doi: 10.1038/nm.2399 21738161 PMC3137275

[39] Park TS Gonzales ER Gidday JM. Platelet-activating factor mediates ischemia-induced leukocyte-endothelial adherence in newborn pig brain. J Cereb Blood Flow Metab. (1999) 19:417–24. doi: 10.1097/00004647-199904000-00007 10197511

[40] Stevens SL Bao J Hollis J Lessov NS Clark WM Stenzel-Poore MP. The use of flow cytometry to evaluate temporal changes in inflammatory cells following focal cerebral ischemia in mice. Brain Res. (2002) 932:110–9. doi: 10.1016/S0006-8993(02)02292-8 11911867

[41] Yilmaz G Arumugam TV Stokes KY Granger DN. Role of T lymphocytes and interferon-gamma in ischemic stroke. Circulation. (2006) 113:2105–12. doi: 10.1161/CIRCULATIONAHA.105.593046 16636173

[42] Yilmaz G Granger DN. Leukocyte recruitment and ischemic brain injury. Neuromol Med. (2010) 12:193–204. doi: 10.1007/s12017-009-8074-1 PMC2878882 19579016

[43] Liesz A Zhou W Mracsko E Karcher S Bauer H Schwarting S. Inhibition of lymphocyte trafficking shields the brain against deleterious neuroinflammation after stroke. Brain. (2011) 134:704–20. doi: 10.1093/brain/awr008 21354973

[44] Deng G Carter J Traystman RJ Wagner DH Herson PS. Pro-inflammatory T-lymphocytes rapidly infiltrate into the brain and contribute to neuronal injury following cardiac arrest and cardiopulmonary resuscitation. J Neuroimmunol. (2014) 274:132–40. doi: 10.1016/j.jneuroim.2014.07.009 PMC4152553 25084739

[45] Seok J Warren HS Cuenca AG Mindrinos MN Baker HV Xu W. Genomic responses in mouse models poorly mimic human inflammatory diseases. Proc Natl Acad Sci U S A. (2013) 110:3507–12. doi: 10.1073/pnas.1222878110 PMC3587220 23401516

[46] Papadimitriou D Xanthos T Dontas I Lelovas P Perrea D. The use of mice and rats as animal models for cardiopulmonary resuscitation research. Lab Anim. (2008) 42:265–76. doi: 10.1258/la.2007.006035 18625581

